# Spontaneous rupture of right gastroepiploic artery aneurysm: a rare cause of hemorrhagic shock. Case report

**DOI:** 10.1590/1516-3180.2017.0070210417

**Published:** 2017-08-21

**Authors:** Talha Sarigoz, Sedat Carkit, Omer Topuz, Tamer Ertan, Ali Koc

**Affiliations:** I Medical doctor and Attending Physician, General Surgery Clinic, Sason State Hospital, Batman, Turkey.; II Medical doctor and Resident Physician, General Surgery Clinic, Kayseri Training and Research Hospital, Kayseri, Turkey.; III Medical doctor and Associate Professor, General Surgery Clinic, Kayseri Training and Research Hospital, Kayseri, Turkey.; IV Medical doctor, Head and Professor, General Surgery Clinic, Kayseri Training and Research Hospital, Kayseri, Turkey.; V Medical doctor and Attending Physician, Radiology Clinic, Kayseri Training and Research Hospital, Kayseri, Turkey

**Keywords:** Gastroepiploic artery, Aortic rupture, Shock, hemorrhagic

## Abstract

**CONTEXT:**

Aneurysms of the gastroepiploic arteries are seen only rarely. They are usually diagnosed during autopsy or laparotomy in patients with hemodynamic instability. Although the operation to treat this condition is relatively easy, delay in making the diagnosis affects the course of the disease.

**CASE REPORT:**

A 57-year-old woman was admitted to the emergency department with abdominal pain and unconsciousness. A computed tomography scan showed extravasation of contrast agent at the head-corpus junction of the pancreas, and the patient underwent exploratory laparotomy under general anesthesia. During laparotomy, aneurysmatic rupture of the right gastroepiploic artery was detected. Control over bleeding was achieved by ligating the right gastroepiploic artery at its origin. The aneurysm was also resected and sent for pathological examination.

**CONCLUSION:**

Especially in cases of unidentified shock, splanchnic artery aneurysms should be kept in mind. Moreover, in the light of the data in the literature, the possibility of death should be taken into account seriously and, if feasible, prophylactic aneurysmectomy should be performed.

## INTRODUCTION

Aneurysms of the gastroepiploic arteries are seen only rarely.^1^ They are usually diagnosed during autopsy or laparotomy in patients with hemodynamic instability.^2^ Although the operation to treat this condition is relatively easy, delay in making the diagnosis affects the course of the disease.^3^

A 57-year-old woman was admitted to the emergency department with abdominal pain and unconsciousness. During laparotomy, aneurysmatic rupture of the right gastroepiploic artery was detected. In this study, the diagnosis and treatment of spontaneous right gastroepiploic artery rupture was discussed in the light of the literature (Table 1).

**Table 1.** Results from search of the literature performed on April 4, 2017

Database	Search strategy	Results	
		Found	Related
MEDLINE (via PubMed)	right AND “gastroepiploic artery”[MeSH] OR “gastroepiploic” AND “artery” OR “gastroepiploic artery” AND “aneurysm”[MeSH] OR “aneurysm”	69	11
Scopus (via Elsevier)	right AND gastroepiploic AND artery AND rupture	44	11
LILACS (via Bireme)	(tw:(right gastroepiploic artery aneurysm))	0	-

## CASE REPORT

A 57-year-old female patient from whom written informed consent was obtained for publication of this manuscript was admitted to the emergency department with a history of abdominal pain of duration two days and slight confusion for the last six hours.

According to the patient’s story, she had a history of atherosclerotic heart disease and hypertension. She was using angiotensin-converting enzyme (ACE) inhibitor, beta blocker and acetylsalicylic acid. She had a history of appendectomy, abdominal hysterectomy, laparoscopic cholecystectomy and incisional hernia operation.

On physical examination, the patient was confused but hemodynamically stable. On abdominal examination, there was diffuse tenderness and involuntary guarding. A rectal examination was unremarkable.

In the blood panel analysis, the white blood cell count was 20.6 x 10^3^/µl, serum hemoglobin concentration 7.2 g/dl, platelet count 342 x 10^3^/µl, hematocrit level 25.8%, creatinine level 4.2 mg/dl and blood urea nitrogen 50 mg/dl. Coagulation tests and other biochemical parameters were normal.

Following examination, resuscitation was started using crystalloid solutions and blood components, and abdominal ultrasound was performed. There was perihepatic, perisplenic and right paracolic free fluid. A computed tomography (CT) scan of the abdomen using intravenous contrast was then performed to search for possible sources of bleeding. In the CT scan, extravasation of contrast agent at the head-corpus junction of the pancreas was observed.

**Figure 1.**
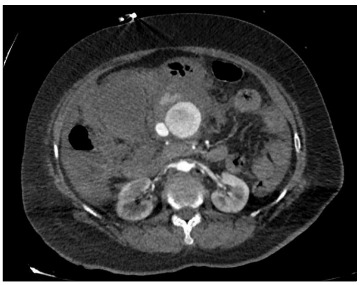
Preoperative late arterial phase computed tomography scan (axial) showing extravasation of intravenous contrast agent.

**Figure 2.**
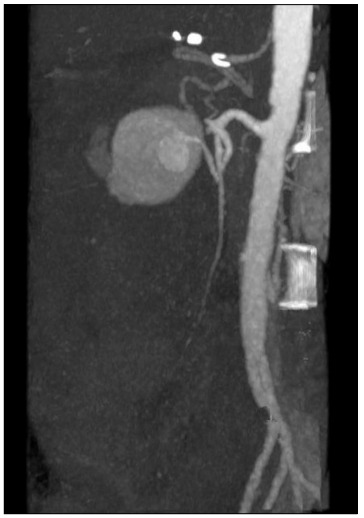
Preoperative computed tomography scan (three-dimensional) showing aneurysmatic rupture.

The patient underwent exploratory laparotomy under general anesthesia (Figures 1 and 2). There was widespread hemoperitoneum and giant hematoma especially in the infrapyloric region. Approximately two liters of fresh blood and hematoma was evacuated from the peritoneal cavity.

The distal part of the stomach was transected using a linear stapler for better exposure. This revealed a ruptured right gastroepiploic artery aneurysm and retroperitoneal hematoma was also seen. Control of bleeding was achieved by ligating the right gastroepiploic artery at its origin. The aneurysm was also resected and sent for pathological examination. The operation was terminated after retrocolic gastrojejunostomy.

**Figure 3.**
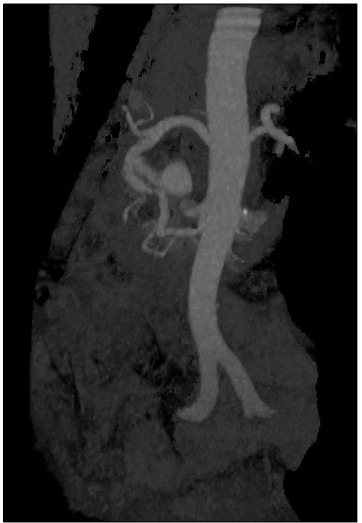
Postoperative computed tomography scan (three-dimensional) showing residual filling of the right gastroepiploic artery.

The CT scan was repeated to confirm that complete resection of the aneurysm and control of concurrent vascular anomalies had been achieved, before discharge (Figure 3). The patient was discharged on the sixth postoperative day without any complications.

## DISCUSSION

Idiopathic spontaneous intraperitoneal hemorrhage, which was first reported by Barber in 1909, was later referred to as abdominal apoplexy by Green and Powers.^4^^,^^5^ It is a rare disease. In the study by Stanley and Zelenoch, 60% of the splanchnic artery aneurysms were reported to originate from the splenic artery, followed by 20% from the hepatic artery, 5.5% from the superior mesenteric artery, 4% from the celiac artery and 3% from the gastroepiploic artery.^6^ In a study conducted by Pulli et al., only one out of 55 patients showed right gastroepiploic aneurysm, and this case was diagnosed incidentally during abdominal ultrasound.^7^ As in the case presented here, right gastroepiploic artery aneurysm is among the rarest forms.

The precise mechanism is not fully known. However, weak tunica media and increased intravenous pressure are thought to be possible causes of rupture. Predisposing factors in the pathogenesis include arteriosclerosis, infection, medial necrosis, trauma, pregnancy and portal hypertension. To confirm the diagnosis, pathological evaluation of the sacrificed specimen is required. In our patient, atherosclerosis was not seen but hypertension was present. Pathological examination of the lesion confirmed that it was a true aneurysm.

The symptoms of splanchnic aneurysm are usually nonspecific. There may be vague abdominal pain before the rupture. After the rupture, noticeable abdominal pain accompanied by hypotension is present. Shimada reported that the symptoms of ruptured and un-ruptured aneurysms differed.^8^ In ruptured aneurysms, upper abdominal pain and hypovolemic shock were present, while in un-ruptured aneurysms, upper abdominal complaints were present. In our case, the patient’s complaints were consistent with a ruptured aneurysm.

A ruptured aneurysm is usually diagnosed through laparotomy in a setting of hemodynamic instability. In less urgent cases, abdominal ultrasound examination, CT scans and angiography are helpful tools for making the correct diagnosis. According to the literature, 81% of gastroepiploic artery aneurysms were diagnosed after rupture.^8^ In Shimada’s study, 44.4% of the patients were diagnosed by means of arteriography, while Morita et al. reported that angiography gave rise to a correct diagnosis rate of 64-76%.^9^

CT scans are the most important imaging test in the emergency setting. With CT scans, intravenous contrast is highly recommended and its effectiveness for locating active bleeding has been proven.^10^ On the contrary, in our study, CT angiography scans did not show the location of the ruptured aneurysm precisely, but because of the extravasation, the patient underwent an operation.

In cases of hemorrhagic shock, the mainstays of treatment are volume resuscitation (either with fluid or with blood components) and control over life-threatening bleeding. In this context, for better exposure and rapid control of bleeding, the distal stomach was transected using a linear cutter stapler, at the expense of losing intactness of the upper gastrointestinal system. This made it easy to reach the pancreatic head and ligate the bleeding ruptured aneurysm. This procedure was undertaken despite lack of mention of this technique in the literature.

Surgical excision, which can be performed through conventional surgery or through laparoscopic surgery, provides complete disease control.^11^ In emergency cases, operative mortality rates have been reported to be around 50-70%, while in elective cases this rate is 0-3%. If possible, the aneurysm should be ligated from the proximal side and the distal portion should be removed. According to the literature, this method is easy and carries low risk.^1^ Transarterial catheter embolization is a non-surgical treatment method and can be performed promptly after angiography in patients with life-threatening hemorrhage. Hemostasis can be achieved easily through this. However, if the radiologist has no experience in this area and if technical difficulties occur, surgical treatment is usually required.

Regarding the follow-up of these patients, there is no consensus in the literature, given that most of the data are from case reports. A control radiological examination can be helpful after resection. If embolization is performed in elderly high-risk patients, observation is recommended.^1^

## CONCLUSION

Especially in cases with unidentified shock, ruptures of splanchnic artery aneurysms should be kept in mind. Moreover, in the light of the data in the literature, the possibility of mortality should be taken into account seriously and, if feasible, prophylactic aneurysmectomy should be performed.
